# iTRAQ-based Proteomic Analysis of Porcine Kidney Epithelial PK15 cells Infected with Pseudorabies virus

**DOI:** 10.1038/srep45922

**Published:** 2017-04-04

**Authors:** Songbai Yang, Yue Pei, Ayong Zhao

**Affiliations:** 1 College of Animal Science and Technology, Zhejiang A&F University, Lin’an, Zhejiang 311300, China

## Abstract

Pseudorabies virus (PRV) is one of the most important pathogens of swine, resulting in severe economic losses to the pig industry. To improve our understanding of the host responses to PRV infection, we applied isobaric tags for relative and absolute quantification (iTRAQ) labeling coupled with liquid chromatography-tandem mass spectrometry to quantitatively identify the differentially expressed cellular proteins in PRV-infected PK15 cells. In total, relative quantitative data were identified for 4333 proteins in PRV and mock- infected PK15 cells, among which 466 cellular proteins were differentially expressed, including 234 upregulated proteins and 232 downregulated proteins. Bioinformatics analysis disclosed that most of these differentially expressed proteins were involved in metabolic processes, cellular growth and proliferation, endoplasmic reticulum (ER) stress response, cell adhesion and cytoskeleton. Moreover, expression levels of four representative proteins, beta-catenin, STAT1, GRB2 and PCNA, were further confirmed by western blot analysis. This is the first attempt to analyze the protein profile of PRV-infected PK15 cells using iTRAQ technology, and our findings may provide valuable information to help understand the host response to PRV infection.

Pseudorabies virus (PRV or suid herpesvirus 1) is a member of the genus Varicellovirus, family Herpesviridae. PRV is the causative agent of Aujeszky’s disease, which can cause neurological and respiratory system disorders in young piglets and death of the fetuses and/or abortion in pregnant sows. Thus, PRV has major economic consequences in pig husbandry^1^,^2^. PRV has a double-stranded DNA genome of approximately 150 kb in length and can infect a broad range of wild and domestic animal species, including ruminants, carnivores and rodents. Pigs are the only natural hosts for PRV^2^,^3^,^4^. Vaccines are widely used to reduce the economic losses caused by PRV infection^5^,^6^. However, in 2012, an unprecedented large-scale outbreak of pseudorabies in pigs in northern and eastern China caused huge economic losses to the swine industry^6^.

The pathogenesis of PRV infection and the interactions between PRV and porcine cells are not fully understood at present^7^,^8^,^9^. Proteomic approaches provide effective tools for facilitating a comprehensive characterization of virus-host interactions, for example, two-dimensional gel electrophoresis (2DE) and matrix-assisted laser desorption ionization-time-of-flight mass spectrometry (MALDI-TOF/MS) approaches have been used to provide the proteomic expression profiles of host cells in response to viral infections, including classical swine fever virus^10^, severe acute respiratory syndrome^11^, H1N1 influenza virus^12^ and spleen and kidney necrosis virus^13^. Stable isotope labeling of amino acids in cell culture (SILAC) has also been used in identifying the differentially expressed proteins of viral infections, including dengue virus type 2^14^, porcine reproductive and respiratory syndrome virus^15^, Enterovirus 71^16^ and herpes simplex virus type 1^17^. These studies have provided extensive insight into understanding the host response to viral infection and have also highlighted the potential antiviral agents capable of targeting the various kinds of viral infection.

Isobaric tags for relative and absolute quantitation (iTRAQ) combined with LC-MS/MS analysis has emerged as a more powerful quantitative proteomic method because this method is more sensitive than traditional proteomic approaches, especially for quantifying low-abundance proteins in the tested samples^18^,^19^,^20^. The iTRAQ-based quantitative proteomic technique has been applied to studies of virus-host interactions, which include porcine circovirus type 2^21^, porcine reproductive and respiratory syndrome virus^22^, porcine epidemic diarrhea virus^23^, bluetongue virus^24^ and transmissible gastroenteritis virus^25^. Until now, the mechanisms of PRV pathogenesis and the interaction between PRV and porcine cells have not been fully understood. It is well known that porcine kidney epithelial cells (PK15) are most widely used for PRV isolation, propagation and basic research. In the present study, we used iTRAQ coupled with LC−MS/MS to identify cellular proteins that were differentially expressed in PK15 cells infected with PRV. The results showed that 466 proteins were significantly changed after PRV infection. These proteins could serve as potential biomarkers of PRV-infected cells and provide new insights into the regulatory mechanism of this disease.

## Results

### Kinetics of PRV propagation in PK15 cells

**Figure 1:**
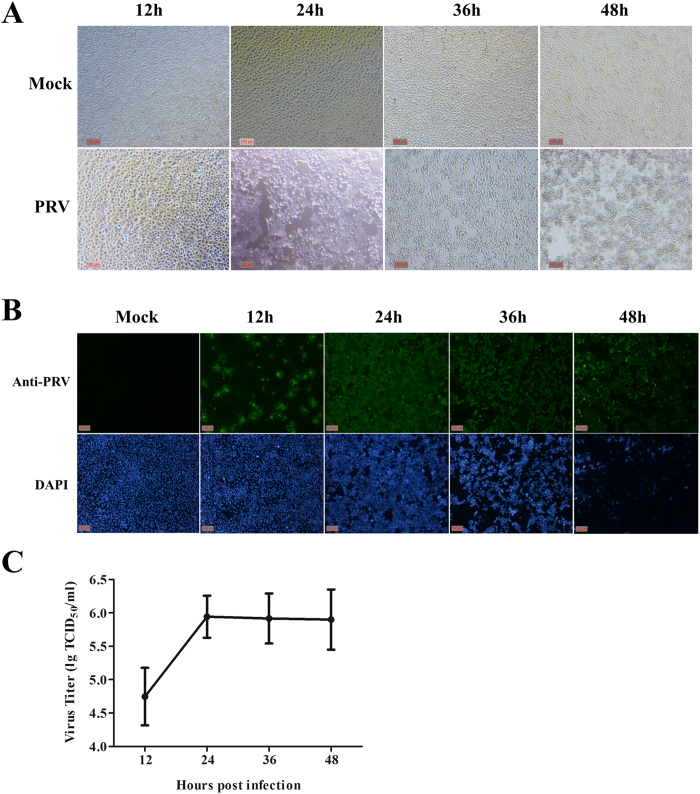
PRV infection in PK15 cells. (**A**) Morphological changes in PK15 cells at 12, 24, 36, 48 h after PRV infection, with mock infected cells as a control. (**B**) Confirmation of the proliferation of PRV by immunofluorescence staining in infected PK15 cells at 12, 24, 36, and 48 hpi, and mock-infected cells at 24 h has a control. (**C**) One-step growth curve of PRV in PK-15 cells.

To determine the kinetics of PRV propagation in PK15 cells and the optimal time-point for proteomic analysis, PK-15 cells were infected with PRV and then monitored for CPE and viral protein expression, in addition to the viral titers, which were detected at 12, 24, 36 and 48 hpi. As shown in Fig. 1A, no obvious CPE was visible at 12 hpi; however, the CPE became readily apparent as the infection progressed. Obvious CPE was observed at 24 hpi and became significant at 36 and 48 hpi, including cell rounding, swelling, granular degeneration of the cytoplasm, cell detachment, and severely diseased cell morphology. The expression of PRV protein was monitored by IFA, and the results showed that almost all cells were infected at 24 hpi (Fig. 1B). The one-step growth curve for PRV in PK-15 cells was also assessed. The viral titer peaked at 24 hpi and then gradually declined (Fig. 1C). Generally, no excessive cytopathic effect of host cells was observed. The time point when viral replication remains high is often regarded as the optimal time for proteomic analysis^23^,^25^. Therefore, PRV- and mock-infected cells were harvested at 24 hpi for further proteomic analysis.

### Protein profile obtained by iTRAQ- coupled LC-MS/MS analysis

To explore the differentially expressed proteins following virus infection, the total proteins of PRV-infected and mock-infected PK15 cells were extracted for iTRAQ coupled with LC-MS/MS analysis. By this approach, a total of 4333 proteins were detected and quantified (Supplementary file 1). A quantitative ratio over 1.5 (fold change >1.5 or <0.67) and a *p* -value < 0.05 were considered differentially expressed proteins. Using the criterion, 234 proteins were significantly upregulated, and 232 proteins were downregulated during PRV infection (Supplementary file 2). Because the current pig genome is poorly annotated compared to the human genome database, there were 52 proteins that remained uncharacterized among the differentially expressed proteins (Supplementary file 2). Therefore, further research is warranted to focus on the functions of these proteins.

### Functional classification of differentially expressed proteins

**Figure 2:**
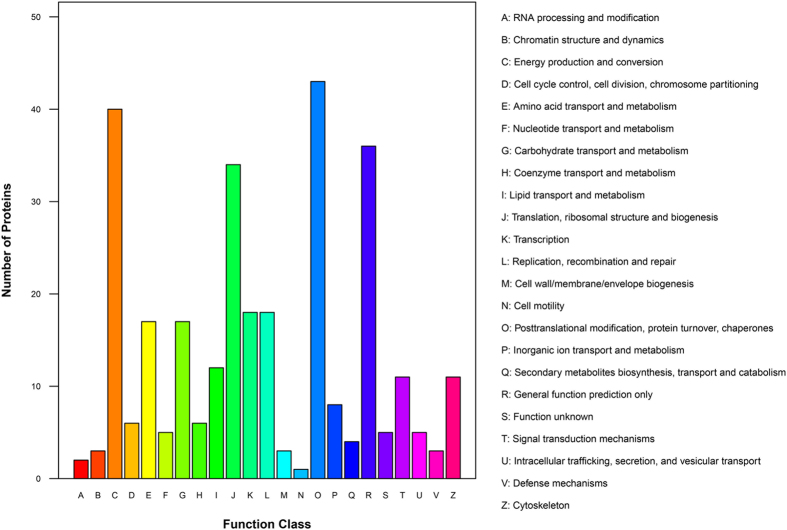
COG function classification of the differentially expressed proteins in PRV infected PK15 cells.

**Figure 3:**
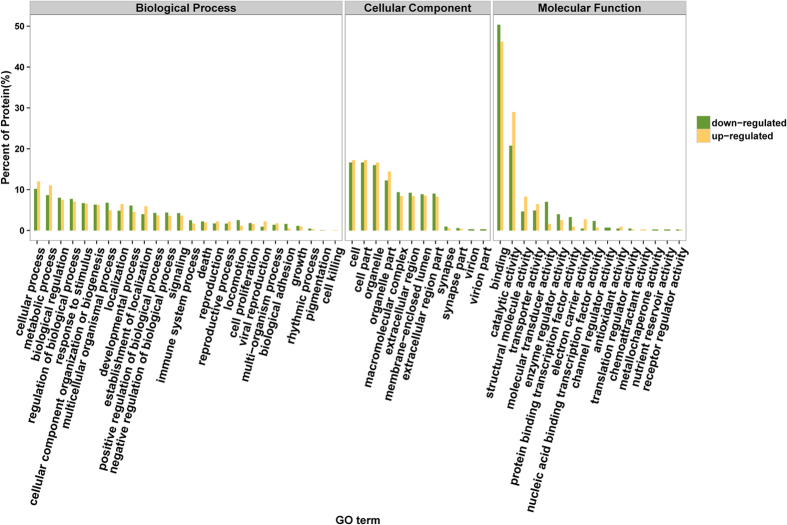
GO analysis of the differentially expressed proteins in PRV infected PK15 cells. Proteins were classified into three main categories: biological process, cellular component, and molecular function. The y-axis indicates the percentage of a specific category of proteins in each main category.

**Figure 4:**
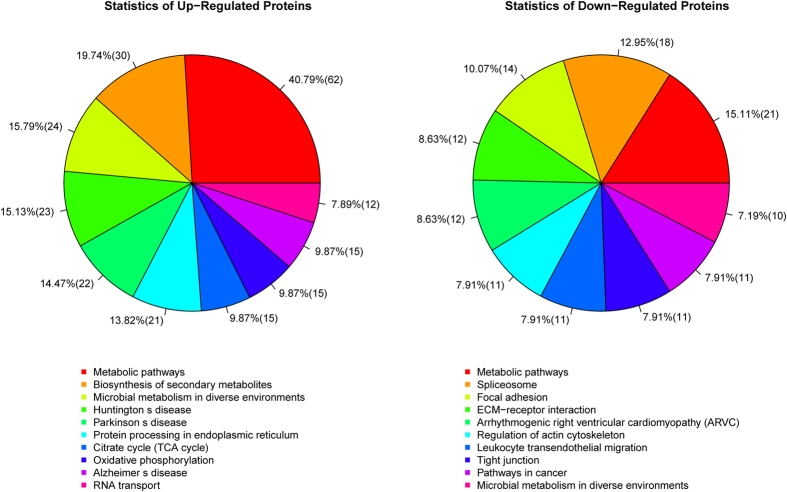
KEGG pathway analysis of the differentially expressed proteins in PRV infected PK15 cells.

These 466 differentially expressed proteins were classified into 23 groups based on their function by COG annotation. The top four groups containing more than 34 proteins were post-translational modification, protein turnover, and chaperones; energy production and conversion; general function prediction only and translation; and ribosomal structure and biogenesis (Fig. 2 and Supplementary file 3). To further extend the molecular characterization of the differentially expressed proteins, the Gene Ontology and UniProt databases were searched, and these proteins were assigned into their different biological processes, molecular functions, and cellular components (Fig. 3 and Supplementary file 4). For biological process annotation, proteins were mainly involved in cellular process, metabolic process, biological regulation, regulation of biological process and response to stimulus. Among the proteins related to metabolic process, tricarboxylic acid cycle was most significantly enriched (*p* = 3.81E-10). The cellular component annotation revealed that these proteins were well distributed in different cell components, and there were 257 differentially expressed proteins, with the most significant *p* value (3.49E-15) being located in the membrane. The major functional categories were binding, catalytic activity, structural molecule activities and transporter activity. In particular, unfolded protein binding (*p* = 5.50E-09) was most significantly enriched (Fig. 3 and Supplementary file 4). The KEGG database was used to identify the pathway involvement of these differentially expressed proteins with most proteins being involved in a metabolic pathway. The upregulated proteins were mainly involved in protein processing in the endoplasmic reticulum and citrate cycle (TCA cycle). The pathways associated with downregulated proteins were related to cell adhesion, ECM-receptor interaction, tight junction, and regulation of actin cytoskeleton (Fig. 4 and Supplementary file 5).

### Validation of protein identification and quantification by western blot

**Figure 5:**
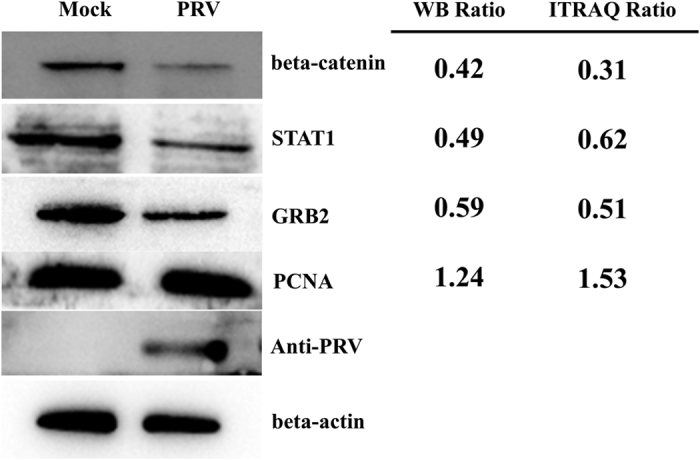
Confirmation of differentially expressed proteins by western blot. Immunoblotting analysis of proteins (beta-catenin, STAT1, GRB2 and PCNA) in PRV-infected or mock-infected PK15 cells. WB ratios and iTRAQ ratios (infection/mock) were shown on the right side. The β-actin protein was used as a control.

To validate the differentially expressed proteins identified via iTRAQ-labeled LC-MS/MS analysis, four proteins (beta-catenin, STAT1, GRB2 and PCNA) based on interest and ratios were selected for analysis by western blot (Fig. 5). The ratios of the four proteins between infected and uninfected cells were consistent with those obtained from iTRAQ approach.

## Discussion

From the perspective of host cell proteins, virus and host cell interactions are highly complex processes often causing numerous changes in the expression of proteins involved in signaling pathways^26^. Currently, various proteomics approaches are widely used to study viral and host cellular interactions^23^,^24^,^27^. PRV can infect PK15 cells with obvious CPEs; therefore, PK15 cells are an appropriate model for the study of PRV infection, with many studies on host - PRV interactions having been carried out on PK15 cells^28^,^29^,^30^. To date, no research has focused on the protein profile of PK15 cells infected with PRV. In this study, iTRAQ combined with LC-MS/MS was used to identify the differentially regulated proteins in PK-15 cells during PRV infection. A total of 4333 proteins were detected and quantified in PRV- and mock-infected PK15 cells, of which 234 were significantly upregulated and 232 downregulated based on a fold change > 1.5 or < 0.67 with *p* value < 0.05 for the differentially expressed proteins. Four representative proteins were verified by western blot analysis and the ratio of infection and mock infection in accordance with iTRAQ results. These differentially expressed proteins were involved in a multitude of biological processes including metabolic pathways, protein processing in endoplasmic reticulum, ECM-receptor interaction, and regulation of actin cytoskeleton. These data could provide clues useful for further analysis of PRV pathogenesis.

The proliferation of virus in cells requires energy and small molecule metabolites, including ATP, NADH, NADPH and the carbon used for the synthesis of nucleotides, lipids, amino acids and carbohydrates^31^. In this study, 83 differentially expressed proteins (17.8%) were classified into metabolic pathways. These proteins were mainly involved in the citrate (TCA) cycle as well as glycolysis and pyruvate metabolism. Fifteen of 17 proteins in the TCA cycle, all 9 proteins in pyruvate metabolism and 8 of 13 proteins in glycolysis were upregulated (Supplementary Figure). These data demonstrate that PRV utilized energy from the host cell to proliferate through the hijacking of host cell metabolic processes. Similarly, as a member of the herpesvirus family, HCMV can activate the TCA cycle and glycolysis simultaneously allowing the carbons from glucose to be delivered to the TCA cycle to produce fatty acids. In contrast, HSV-1 activates pyruvate carboxylase to induce pyrimidine biosynthesis^32^.The mechanisms of virus-host metabolic interplay warrant further study, especially in how the key genes involved in metabolic pathways can regulate viral infection, because these studies provide novel targets for antiviral drug discovery through metabolic pathway inhibitors. The key enzymes in metabolic pathways that were upregulated after PRV infection are involved in the gene expression regulation system; therefore, incorporating systems analysis, including transcriptomic^33^, proteomic^8^, and metabolomic data, could help to gain a better understanding of this specific mechanism.

The endoplasmic reticulum (ER) is critical for protein synthesis and maturation. ER reside on many molecular chaperones that assist protein folding and assembly^34^. Numerous studies show that viral infections can alter endoplasmic reticulum (ER) and activate the unfolded-protein response (UPR), thereby facilitating viral replication^35^,^36^,^37^,^38^. In the current study, 21 of the differentially expressed proteins were involved in protein processing in the endoplasmic reticulum. Interestingly, all of the 21 proteins were upregulated (Supplementary Figure). Among these were the heat shock proteins hsp 90-beta (hsp90ab1), 90 kDa beta member 1(hsp90b1), 70 kDa protein 5(hspa5) and 105 kDa (hsph1). Similarly, hsp27 was also significant altered at 4 h of PRV infction in Madin-Darby bovine kidney cells using SILAC mass spectrometry methods, since only a small number of host cell proteins changed during early stages of PRV infection, other stress-related proteins did not show significant variations^7^,^8^. Heat-shock proteins can facilitate protein folding, heat-shock response activation might be a virus-specific function ensuring proper protein synthesis and; therefore, ER stress proteins may also be important for virus replication^37^,^39^,^40^. Hsp90 is involved in the assembly and nuclear transport of viral RNA polymerase subunits and facilitates viral replication in HIV-1, Ebola virus, hepatitis B virus, hepatitis C virus, and Rotavirus^41^,^42^,^43^,^44^,^45^,^46^. Previous research has shown that hsp90 can interact with acetylated α-tubulin to promote nuclear transport of HSV-1 capsid protein and interact with the HBV reverse transcriptase to facilitate the formation of a ribonucleoprotein (RNP) complex that is required early in replication^47^,^48^. Thus, hsp90 can be targeted using inhibitors to resist viral infections^49^,^50^. Similarly, hspa5 is an essential target for a viral infection, such as Ebola virus^51^. Some other upregulated ER-stress proteins, such as calreticulin and calnexin, that are important for calcium storage and protein folding^52^, also play an important role in viral infections. Calreticulin is induced during HBV infection and can enhance HBV replication by antagonizing the IFN pathway^53^. Calnexin can directly bind to mature S protein and result in conferred infectivity of severe acute respiratory syndrome coronavirus^54^. Thus, these upregulated proteins may also play an important role in PRV replication, and further research is required to investigate the function of these proteins during PRV infection.

Numerous ribosomal proteins and translation elongation factors were upregulated during PRV infection (Supplementary file 2). We speculate that PRV would utilize the host cell protein synthesis system to produce a large number of viral proteins after entry into the cell. Among these proteins, 60 S acidic ribosomal protein P0 (RPLP0) was detected and showed significant changes in the early stages of infection by PRV in Madin-Darby bovine kidney cells^8^. Several DNA helicase complexes required for the process of DNA replication including the minichromosome maintenance protein (MCM2, MCM4 and MCM6) were downregulated in the PRV-infected cells. Similarly, nuclear accumulation of MCM4 and MCM6 was reduced in HSV-1-infected human epithelial larynx carcinoma HEp-2 cells^55^. MCM4, MCM6, and MCM7 subunits bind tightly with MCM2, causing a reduced affinity to form the MCM core complex that acts as a DNA helicase in the unwinding of cellular dsDNA^56^,^57^. Herpesviruses including PRV contain their own helicases essential for the formation and elongation of the replication fork in viral replication^1^,^58^,^59^,^60^. Therefore, we speculate that PRV hijack cellular components involved in host cell replication and promote viral genome replication. However, another critical host replication factor proliferating cell nuclear antigen (PCNA) was upregulated in PRV-infected cells. PCNA is a clamp that acts as a processivity factor in DNA replication and is required for HSV-1 replication and histone deposition^61^,^62^. Taken together, these data indicate that PRV targets multiple proteins involved in host cell replication and translation, and the function of these proteins in PRV infection needs to be further investigated.

Heterogeneous nuclear ribonucleoproteins (hnRNPs) are a family of RNA-binding proteins present in the cell nucleus that are known for their role in pre-mRNA splicing^63^. Alphaherpesviruses can lead to drastic changes in RNA metabolism through their herpesviral shutoff mechanisms including modulation of hnRNPs. The host shutoff protein pUL41 of HSV-1 can preferentially degrade mRNA containing AU-rich elements^8^,^64^. In addition, unlike the numerous cellular pre-mRNA, HSV-1 genes were largely unspliced and evolved a number of strategies to inhibit host cell splicing^55^,^65^. Previous studies have shown that the expression levels of hnRNPs were affected by HSV-1or PRV infection using SILAC and 2-DE mass spectrometry approachs^8^,^17^,^55^. Here, eight hnRNPs family members (A2B1, A3, C, K, M, R, U and UL2) were also identified and shown to be downregulated after PRV infection (Supplementary file 2). Some of these proteins have known roles in other viral infections. For example, hnRNPU is a potential HIV restriction factor^66^. hnRNPA2/B1 interacts with influenza A viral protein NS1 and inhibits viral replication^67^. Therefore, we hypothesized that the decreased expression of these proteins may play an important role in the replication of PRV.

Cytoskeleton proteins play a critical role in the maintenance of cell morphology, cell movement, and cell-to-cell attachment. Many viral infections cause host cell cytoskeletal disruption or disorganization^68^,^69^,^70^. In our study, numerous proteins involved in cytoskeleton networks and cell communication were altered after PRV infection. Among them, actin alpha 4 (ACTN4), acts as an actin-binding and cross-linking factor, is essential for a number of important cellular functions including cell adhesion and signal transduction and can interact with nucleoprotein to facilitate influenza A viral infection^71^. Other transmembrane receptors involved in cell-cell and cell-extracellular matrix (ECM) interactions are integrin beta 1and integrin beta 4. 12 of 13 proteins involved in the ECM-receptor interaction pathway were downregulated after PRV infection. After PRV infection, at 24 hpi, we observed significant cell pathological changes with high viral titer. Therefore, we inferred that the host cell cytoskeletal and ECM-receptor interaction disruption may contribute to PRV proliferation and release.

Viruses can apply various strategies to suppress the host immune system after infection. STAT1 plays a critical role in the JAK/STAT pathway involved in mediating the cellular interferon response^72^. In response to IFN-γ stimulation, STAT1 and STAT3 homo- and heterodimers bind to IFN-γ activated sequence (GAS) elements^73^. Our proteomic data show that STAT1 and STAT3 are decreased with PRV infection. These data suggest that PRV suppresses canonical interferon signaling through the JAK-STAT1 pathway. Another signaling protein, β-catenin, involved in Wnt/β-catenin signaling, a canonical pathway known to play a vital role in numerous cellular activities was also decreased after PRV infection. Previous research has shown that β-catenin can act as a host restriction factor to repress basal HIV transcription in astrocytes^74^,^75^. However, these proteins need to be further studied to see whether they play an important role in PRV infection.

In conclusion, this study provides a comprehensive analysis of the proteomics profile of PRV-infected PK-15 cells through ITRAQ-based quantitative proteomics. A total of 466 significantly changed proteins were identified; however, the function of these differentially expressed proteins remains mostly descriptive. These proteomics results are preliminary data that require further investigation to understand the roles of these proteins in PRV infection, thereby enabling new antiviral therapeutic targets of PRV infection.

## Methods

### Cell culture and virus infection

The virulent wild-type PRV strain PRV ZJ (Zhejiang) was utilized in this study^76^. PK15 cells (obtained from the American Type Culture Collection, Manassas, VA) were grown in modified Eagle’s medium (MEM, Gibco, Life Technologies, Austin, TX) supplemented with 10% fetal bovine serum (Gibco, Life Technologies, Austin, TX) and maintained in a humidified incubator at 37° C and 5% CO2.

The monolayer of confluent PK15 cells was dispersed with 0.25% trypsin and 0.02% ethylenediaminetetraacetic acid (EDTA) and seeded in 6-cm cell culture flasks. PK15 cells were cultured for nearly 24 h for 80% confluence and washed twice with PBS. Then, the cells were infected with PRV with 70 μL of 10^5.67^/mL 50% tissue culture infective dose (TCID50) per well. After 1 h of adsorption, infected cells were maintained in MEM supplemented with 2% FBS. Uninfected PK15 cells were used as the mock-infected group. The PRV-or mock-infected cells were collected at 24 h postinfection (hpi). Each group was processed with three independent biological replicates. Viral propagation was confirmed with cytopathic effect (CPE) under a light microscope and one-step growth curve of PRV at 12, 24, 36, and 48 hpi.

### Immunofluorescence assays

The cells infected with PRV at different time points and mock-infected cells at 24 h were washed twice with PBS and then fixed with 4% paraformaldehyde for 20 min at room temperature (RT) and permeabilized with 0.2% Triton X-100 (T8200, Solarbio life science) for 15 min. The cells were then incubated with a blocking buffer (PBS containing 5% bovine serum albumin [BSA]) at RT for 30 min. After three washes with PBS, the cells were stained with anti-PRV rabbit antibody (PA1–081, Thermo Fisher Scientific) at RT for 1 h. After being washed with PBS, the cells were incubated with Alexa Fluor 488 conjugated goat anti- rabbit antibody (A-11008, Thermo Fisher Scientific). The nuclei were stained with DAPI (C0060, Solarbio life science).

### Protein isolation, digestion, and labeling with iTRAQ reagents

Infected and mock-infected PK15 cells were washed twice with PBS and then collected using cell scrapers after the addition of 200 μl TEAB (0.5 M triethylammonium bicarbonate) dissolution buffer. The samples were broken by the ultrasonic wave for 15 min, and then following centrifugation at 12000 r/min for 20 min, the supernatant was subsided by adding 4-fold volume of cold acetone containing 10 mM DTT for approximately 2 h. After centrifugation at 12000 r/min for 20 min at 4 °C, the precipitate was collected and mixed with 800 μl cold acetone at 56 °C to break the proteins’ disulfide bonds. Following centrifugation at 12000r/min for 20 min at 4 °C, the dried precipitate was collected and dissolved with 100 μl TEAB dissolution buffer. The protein concentration was determined using the Bradford protein assay.

An aliquot of total protein (100 μg) was dissolved to 100 μl in a dissolution buffer and then diluted with 500 μl 50 mM NH4HCO3. 2 μg trypsin was added and then incubated overnight at 37 °C. After protein digestion, equal volume of 0.1% formic acid was added for acidification. Peptides were purified on Strata -XC18 pillar, which was first activated with methanol, then balanced by adding 1 ml 0.1% formic acid three times, washed with 0.1% formic acid + 5% acetonitrile two times, and eluted with 1 ml 0.1% formic acid + 80% acetonitrile. The peptides were dried by vacuum centrifugation. The dried peptides powder was redissolved with 20 μl 0.5 M TEAB for peptides labeling.

The peptides were labeled with iTRAQ Reagent-8 plex Multiplex Kit (AB Sciex U.K. Limited) according to the manufacturer’s instructions. The samples and labeled marker were as follows: PRV-infected samples were labeled with iTRAQ tag 115, iTRAQ tag 116, or iTRAQ tag 117, and mock-infected samples were labeled with iTRAQ tag 118, iTRAQ tag 119, or iTRAQ tag 121. All of the labeled samples were mixed with an equal amount. The labeled samples were fractionated using high-performance liquid chromatography (HPLC) system (Thermo DINOEX Ultimate 3000 BioRS) using a Durashell C18(5 μm, 100 Å, 4.6 × 250 mm). Finally, 12 fractions were collected.

### LC-MS/MS Analysis

Data acquisition was performed with a Triple TOF 5600 System (AB SCIEX, Concord, ON). Samples were chromatographed using a 90 min gradient from 2–30% (mobile phase A 0.1% (v/v) formic acid, 5% (v/v) acetonitrile; mobile phase B 0.1% (v/v) formic acid, 95% (v/v) acetonitrile) after direct injection onto a 20 μm PicoFrit emitter (New Objective) packed to 12 cm with Magic C18 AQ 3 μm 120 Å stationary phase. MS1 spectra were collected in the range 350–1,500 m/z for 250 ms. The 20 most intense precursors with charge state 2–5 were selected for fragmentation, and MS2 spectra were collected in the range 50–2,000 m/z for 100 ms; precursor ions were excluded from reselection for 15 s.

### Data analysis

The original MS/MS file data were submitted to ProteinPilot Software (version 4.5, AB Sciex) for data analysis. MS/MS data were searched against Sus scrofa UniProt database (March 9, 2016, containing 35,303 sequences, http://www.uniprot.org/proteomes/UP000008227). The following search parameters were used: the instrument was TripleTOF 5600, iTRAQ quantification, cysteine modified with iodoacetamide; biological modifications were selected as ID focus, the Quantitate, trypsin digestion, Bias Correction and Background Correction was checked for protein quantification and normalization. For false discovery rate (FDR) calculation, an automatic decoy database search strategy^77^ was used to estimate FDR using the PSPEP (Proteomics System Performance Evaluation Pipeline Software) algorithm. Only proteins with at least one unique peptide and unused value more than 1.3 were considered for further analysis. Among the identified peptides, some were excluded from the quantitative analysis for one of the following reasons. (1) The peaks corresponding to the iTRAQ labels were not detected. (2) The peptides were identified with low identification confidence. (3) The peptides were claimed by more than one protein. (4) The S/N (signal-to-noise ratio) for any peptide ratio was too low. (5) Peptides had a combined feature probability < 30% because of semitryptic peptides, peptides missing an iTRAQ reagent label, peptides with low probability modifications and peptides with large delta masses. For protein abundance ratios measured using iTRAQ after normalization, we specifically used ratios with *p* value < 0.05, and only fold changes > 1.5 or < 0.667 were considered significant.

### Bioinformatics analysis

The identified and differentially expressed proteins sequences were mapped with Gene Ontology Terms (http://geneontology.org/). A homology search was first performed for all the identified sequences with a localized NCBI BLASTP program against NCBInr animal database. The e-value was set to less than 1e-5, and the best hit for each query sequence was taken into account for GO term matching. The GO term matching was performed with blast2go v4.5 pipeline^78^. Clusters of Orthologous Groups of Proteins System (COG, http://www.ncbi.nlm.nih.gov/COG/) was employed for the functional annotation of genes from new genomes and for research into genome evolution. Pathway analyses were conducted using the Kyoto Encyclopedia of Genes and Genomes (KEGG) platform. The pathway enrichment statistics were performed by Fisher’ s exact test, and those with a corrected *p* value < 0.05 were considered the most significant pathways.

### Western blot analysis

The infected and mock-infected cells were collected at 24 hpi. Equivalent amounts of cell lysates from each sample were mixed with 5 × sample loading buffer and boiled for 10 min, separated by 12% SDS-polyacrylamide gels and transferred to PVDF membranes (Millipore). The membranes were blocked with 5% nonfat milk in Tris-buffered saline containing 0.1% Tween-20 (TBST) and then incubated overnight at 4 °C with primary antibodies specific for β-Actin (4967, Cell Signaling Technology), beta-catenin (51067-2-AP), STAT1 (10144-2-AP), GRB2 (10254-2-AP) and PCNA (60097-1-Ig) purchased from Proteintech Group. Membranes were then washed with TBST three times and incubated with a horseradish peroxidase (HRP) conjugated secondary antibody (Proteintech Group) for 1 h at ambient temperature. Finally, protein bands were visualized by addition of the SuperSignal West Pico chemiluminescent substrate (Thermo, Rockford, IL) reagent.

## Additional Information

**How to cite this article**: Yang, S. *et al*. iTRAQ-based Proteomic Analysis of Porcine Kidney Epithelial PK15 cells Infected with Pseudorabies virus. *Sci. Rep.* **7**, 45922; doi: 10.1038/srep45922 (2017).

**Publisher's note:** Springer Nature remains neutral with regard to jurisdictional claims in published maps and institutional affiliations.

## Supplementary Material

Supplementary Information

Supplementary File 1

Supplementary File 2

Supplementary File 3

Supplementary File 4

Supplementary File 5
