# Supplementary material for: iTRAQ-based Proteomic Analysis of Porcine Kidney Epithelial PK15 cells Infected with Pseudorabies virus
Source: Sci Rep. 2017 Apr 4;7:45922. doi: 10.1038/srep45922 (PMC5379687; doi:10.1038/srep45922)

iTRAQ-based Proteomic Analysis of Porcine Kidney Epithelial PK15 cells Infected with Pseudorabies virus

Songbai Yang[#], Yue Pei[#], and Ayong Zhao[*]

College of Animal Science and Technology, Zhejiang A&F University, Lin'an, Zhejiang 311300, China

Supplementary file 1 Total proteins (4333) identified and quantified by iTRAQ.

Supplementary file 2 Differentially expressed proteins (466) identified under certain filter criteria.

Supplementary file 3 COG function classification of the differentially expressed proteins.

Supplementary file 4 GO enrichment analysis of differentially expressed proteins.

Supplementary file 5 KEGG analysis of differentially expressed proteins.

Supplementary figure Significance statistically analysis of KEGG pathways of differentially expressed proteins.

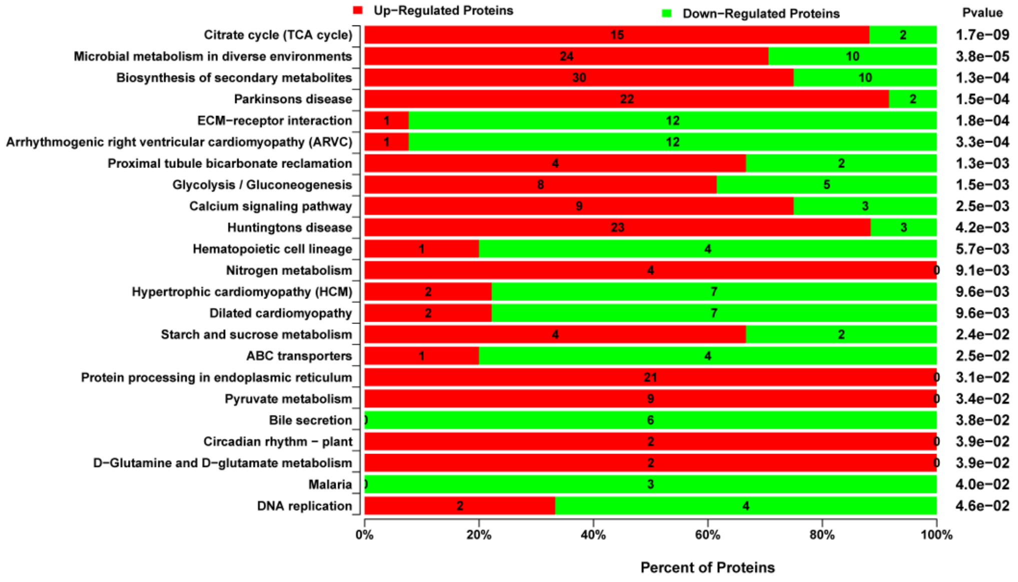

Supplement: Supplementary Information [file srep45922-s1.pdf]
